# Sebaceous horn: a rare clinical image

**DOI:** 10.11604/pamj.2023.45.171.41153

**Published:** 2023-08-21

**Authors:** Rajiv Sonarkar, Avinash Rainait

**Affiliations:** 1 Department of Surgery, Datta Meghe Medical College, Nagpur Datta Meghe Institute of Higher Education and Research (DU), Sawangi, Wardha, India

**Keywords:** Sebaceous horn, sebaceous cyst, keratoacanthoma

## Image in medicine

Sebaceous horn, also known as a cutaneous horn, is a relatively rare skin lesion that typically appears as a cone-shaped, hard, and keratinous growth on the skin's surface. While it can occur on various body parts, it most commonly appears on the face, ears, nose, and back of the hands. This image shows a sebaceous horn on the lateral aspect of the left thigh, a rare place to occur. The first step in diagnosing a sebaceous horn is a thorough clinical examination of by a clinician. They will visually be inspecting the lesion, noting its size, shape, colour, and texture. A detailed medical history from the patient may include information about the lesion's duration, any changes over time, and associated symptoms such as pain, itching, or bleeding. Dermoscopy can help identify specific features, such as vessels, scales, and other characteristics, that aid in diagnosing sebaceous horns and ruling out other conditions If there is uncertainty about the diagnosis or if the sebaceous horn is large, has irregular features, or shows any signs of malignancy, a biopsy may be recommended. Sebaceous horns can sometimes resemble other skin conditions or tumors, including actinic keratosis, squamous cell carcinoma, verruca vulgaris, or seborrheic keratosis. Therefore, it is important to consider these possibilities and use the diagnostic tools mentioned above to differentiate sebaceous horns from other lesions. Once the diagnosis is confirmed, the treatment plan can be developed. Treatment options for sebaceous horns may include surgical excision, cryotherapy, and laser therapy.

**Figure 1:**
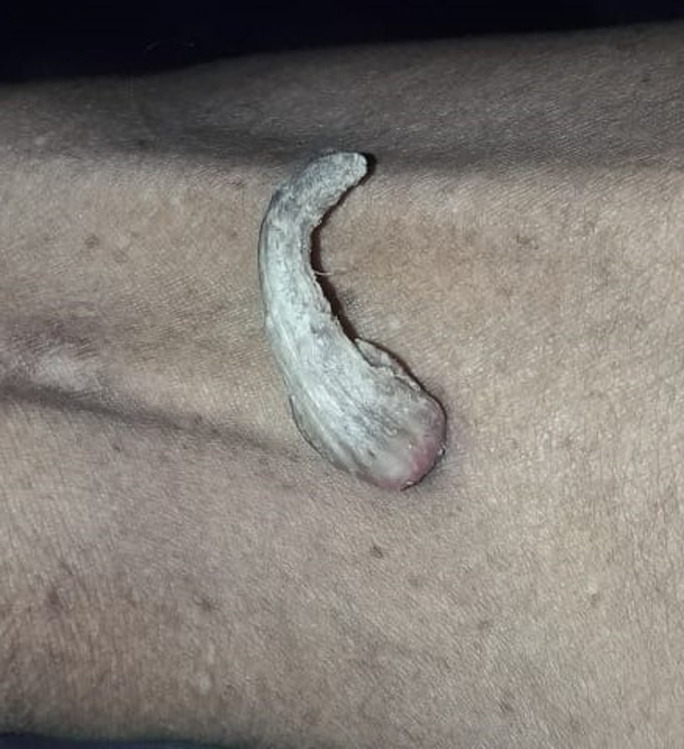
sebaceous horn on the lateral aspect of the left thigh

